# On the Decomposition of Aliment

**Published:** 1822-03

**Authors:** 

**Affiliations:** Liverpool.; Liverpool.


### PHYSIOLOGY.

### ART. IV.—*On the Decomposition of Aliment.* By Drs. SILLAR and HOOD, Liverpool.

THE diversity of opinion that prevails on indigestion, still invites to experiment. We take up the subject with the experiments of REAUMEUR, published in the Memoirs of the Royal Academy of Paris, for 1752. He put pieces of flesh into tubes, open at both ends, for the purpose of admitting the free access of the gastric juice: birds of prey were forced to swallow the tubes thus prepared; and, the pieces of flesh having, in all his experiments, undergone some dissolution, he conceived that this change had been effected by the solvent powers of the gastric juice. Reaumeur made similar experiments on birds with gizzards; but, in place of the pieces of flesh, he substituted grain, which was also dissolved in its passage through the intestinal canal. This solution, he also supposed, was chiefly caused by the gastric juice.

He, likewise, made two attempts to dissolve meat out of the body; but, in both, he completely failed. It was from the experiments of Reaumeur that Haller concluded, "that the gastric juice, even with the aid of heat, does not digest out of the body."

After the two memoirs of Reaumeur came that of Mr. HUNTER, in the Philosophical Transactions for 1772. In a few instances of sudden death, he found parts of the stomach in a half-dissolved state, which he supposed to have been occasioned by the gastric juice operating on the stomach itself after death. On Mr. Hunter's veracity we have implicit faith; but, while we give full credit to his facts, we must beg leave to dissent from his explanation of their cause. On this subject, however, we shall have occasion to make a few remarks hereafter.

The Abbé SPALLANZANI repeated the experiments of Reaumeur, and varied them to satiety; but his trials of artificial digestion were rather more successful than those of his predecessor.

We extract from Spallanzani's work two of his experiments, the most favourable to the theory which he supports.

"I exposed to the sun two phials, filled, to a certain height, with gastric juice from crows; in one of which were immersed several pieces of beef, and, in the other, crumbs of wheaten bread. Nine hours of sunshine much forwarded the artificial digestion, which was the object of my inquiry. A good part of the flesh was reduced to a kind of glue, that, when handled, adhered to the fingers: nothing like flesh remained in any of the pieces, but the nucleus, which was still consistent and fibrous; which two qualities it lost next day. After having been exposed to the sun six hours, the nuclei, like the outside, no longer retained a fibrous structure. In the sun, the heat, as well on the first as the second day, was between 122° and 135° of Fahrenheit's thermometer. The gastric liquor produced upon the bread a change analogous to that which the flesh had undergone. It had not only lost its white colour, but had become viscous, and no longer presented to the eye the appearance, though it retained somewhat of the taste, of bread. Of bread, as well as flesh, immersed in water, and exposed to the sun for the same time, there was a perceptible diminution; but it was inconsiderable when compared with that produced by the gastric juice."—*Dissertation* ii. p. 95.

"I took two tubes, sealed hermetically at one end, and at the other with wax: into one I put several bits of mutton, and into the other several bruised grains of wheat, and then filled them with gastric liquor; and, as the warmth of the stomach is probably another condition necessary to the solution of food, I contrived to supply it, by fixing the tubes under my axillæ. In this situation I kept them, at different intervals, for three days, at the expiration of which time I opened them. The tube with the grains of wheat was first examined: most of them now consisted of the bare husk, the flour having been extracted, and forming a thick grey sediment. The flesh, in the other tube, was, in a great measure, dissolved, and was incorporated with the gastric juice, which had become more turbid and dense. What little remained had lost its redness, and was now exceedingly tender. Upon putting it into fresh gastric liquor, the remainder was dissolved in the course of a day."—Spall. 57.

These are the strongest proofs which Spallanzani has been able to adduce in favour of solution by the gastric fluid out of the body.

Let us contrast these results with the facts known in healthy digestion. A period, varying from four to six hours, completes the digestion of a full meal: four days, with a change of gastric fluid, are required to effect the solution of a few pieces of mutton out of the body. The gastric juice being the same in both cases, the difference in the rapidity of solution must, therefore, depend on the receptacle, and not on the solvent.

Taking these experiments of Spallanzani exactly as they are given, they only prove that the gastric juice is a feeble agent in the digestive process; and the following experiment, by the same author, leads the mind anew to the inquiry for some other influence.

"Having prepared several glass tubes, of the length of six inches, I sealed them hermetically at one end, and the opposite extremities were drawn out so as to form elongated cones. Through the open ends of these cones, I poured a quantity of gastric fluid, together with a few pieces of flesh. I then introduced the cones, by the bases, into the stomachs of some crows, allotting one to each bird; and, when they rested upon the bottom of the stomachs, their apexes came out at the mouth. The flesh remained several hours immersed in the gastric fluid, without showing any sign of decomposition." But, "when the cones were kept ten or twelve hours in the stomach, the flesh was, generally, reduced to a coloured gelatinous pulp."—SPAL. 99.

The late Dr. MONTEGRE, of Paris, who died at St. Domingo, possessed the faculty of vomiting voluntarily, and made, a few years back, repeated experiments with his own gastric juice, vomited in the morning before breakfast; but, all his efforts to dissolve food in it proving abortive, he alleged that the gastric juice is only a bland diluent, like saliva. This opinion has received the support of Professor THENARD, who favours it in his public lectures.

After mature consideration of all the experiments and authorities, for and against the solvent powers of the gastric juice, we confess to have felt greatly disposed to coincide with the opinions of Thenard and Montegre. Besides, the stomach is not the only part of the body in which decomposition takes place. HELVETIUS and SPALLANZANI state, that flesh and fish have been found, half decomposed, in the gullets of birds; and Dr. CURRIE, of this place, kept a gentleman alive, from the 17th of October to the 6th of December, by nutritive injections.

Assimilation and secretion, also, evidently demonstrate, that the vital force is possessed of decomposing properties. With these reasonings in view, the following experiments were executed:

1st. A piece of roasted mutton, slightly powdered with salt, was put into the rectum of a dog. It was extracted at the expiration of eleven hours, and found changed, on the whole of the outside, into a whitish-brown saponaceous paste. A small part of the centre retained its fibrous appearance.

2d. Two pieces of roasted veal were introduced into the rectum of the same dog; one of the pieces being enclosed in a single fold of muslin. They were allowed to remain sixteen hours. The piece without the muslin was pulverulent externally, and seemed only to require the aid of moisture to have the appearance of paste: its centre was unaltered. The piece contained in muslin exhibited nearly the same appearance; but its pulverulence did not extend so far towards the centre as in the other piece. This experiment succeeded less perfectly than the first: a thick layer of feces coated the veal, and, doubtless, sheltered it from the mucus of the rectum and the vital force.

We repeated the first experiment, using veal instead of mutton; and, the result being the same, it is unnecessary to detail it.

3d. A dog, that had fasted twelve hours, was killed. Two drachms of gastric juice were obtained from the stomach, and poured into a small oval phial, containing a cylinder of boiled beef, weighing five grains. The mouth of the phial was closed with a ground stopper, firmly secured by a silver top screwed upon it; and, to obtain the heat of the body, the phial was placed in the rectum of another dog, where it remained eleven hours. About three-quarters of the cylinder of beef were dissolved; the remainder was blackish and viscous.—This experiment quite convinced us, that the gastric juice, when obtained pure, is slightly solvent, even out of the body, if the artificial digestion be managed with the necessary precautions.

We repeated the last experiment; but, in place of gastric juice, the phial was half-filled with saliva. The piece of beef, which was put into it, underwent no perceptible change, but its smell was very offensive.

We tried to digest boiled beef in gastric juice, in a temperature varying from 50° to 60° of Fahrenheit; but did not succeed. The beef, however, had no unpleasant smell when taken from the phial.

4th. An incision was made in the thigh of a dog, separating the cellular membrane from the muscles, and a slice of boiled mutton put into the wound, which was then closed with a stitch. The wound was examined at the expiration of thirteen hours. Its edges had united by the first intention; so that we were obliged to use the scalpel to get out the mutton, which was found partly decomposed, and partly fibrous.

5th. On extracting the mutton, another piece was introduced into the same wound, with the intention of permitting it to remain twenty-four hours; but, on the expiration of seven hours, the pulp of the mutton was observed escaping through the lips of the wound; and, on examination, it was discovered that the whole of the mutton was already decomposed into a soft mass, in which no trace of fibrous structure could be detected.

The difference between these two experiments may be explained in this manner: the vitality of the wound becomes increased by the subsequent inflammation.

6th. A small slice of raw flesh was allowed to remain twelve hours in the wound: the side applied to the muscles of the dog was covered with a stratum of pulp; the whole had a parboiled appearance, and was very tender.

The slices of flesh used in these experiments weighed from fifteen to twenty grains. The decomposition appeared most successful when the meat was cut in the direction of its fibres. It may also be observed, that, when meat is put into a fresh wound, adhesion is apt to ensue between the living and dead parts, and no decomposition is effected where adhesions are formed.

These experiments do not permit us to deny that the gastric juice exercises power as a solvent. But we are authorized, at the same time, to conclude, that its agency is only of secondary importance in the decomposition which takes place in the stomach of animals. As a secretion, it is scanty: as a solvent, it requires co-operation.

We would argue, that the vital action of the stomach is the chief cause of digestion; or, that wherever there are nerves and blood-vessels, that there also must exist a decomposing power. After a full meal, the inner surface of the stomach is in close contact with the alimentary mass, from the whole periphery of which decomposition begins, and advances towards the centre. The decomposed aliment mingles with the drink and the gastric juice, in which it is partly dissolved, and partly floats: this mixture forms the chyme, which is gently forwarded through the pylorus by the contraction of the stomach. As the expansion of the stomach is caused by the alimentary mass, its inner surface does not cease to be in constant contiguity with it, notwithstanding the diminishing bulk of the aliment. When, however, the quantity of food is inconsiderable, its upper surface is not in contact with the stomach when the body is in an erect position: hence, the duration of digestion is not in exact proportion to the quantity of aliment. A moderate quantity of wine, or diluted spirits, augments the vital force of the stomach, and, consequently, increases its digestive power.

Digestion proceeding more rapidly in repose, does not lead us to infer, that the decomposing power of the stomach is lessened by exercise; but violent exercise prevents that determination of blood to it, which should follow repletion: the secretion of the gastric juice must, consequently, be less abundant, and the liquids taken with the food speedily absorbed, to supply the waste by sensible perspiration. The presence of much food in the stomach, when its vital action is high, is, likewise, very apt to throw it into a state of spasm, by which its contents are most frequently ejected.

The stomach retains the power of decomposing substances for some time after pulsation and respiration have ceased. "I made," says Spallanzani, "the experiment upon a raven that had been dead for an hour, and had now only the temperature of the atmosphere. Forty-two grains of beef, cut into pieces, were forced into the stomach, which was opened after the bird had been seven hours exposed to the sun ; and here, instead of solid flesh, I found only the usual pulpy mass, partly in the stomach, and partly in the duodenum." Spallanzani regarded this as a fine instance of solution by the gastric juice ; but, the beef being found partly in the duodenum, evidently shows that the stomach had acted upon it.

Respecting the actual amount of solution effected by the gastric juice in digestion, we can concede no more influence to it than what it possesses out of the body.

The most rapid and effectual solution of meat in the gastric juice, which we know on record, is that effected by Dr. Stevens; who, in the space of eight hours, dissolved twelve grains of beef in half-an-ounce of gastric juice, exposed to a heat of 102°. It must, therefore, be obvious, that, if digestion depended solely on the gastric juice, the process would not only be tedious, but the quantity of the solvent fluid required would, also, be enormous.

When autopsies of the intestinal canal are performed with care, nothing is more common than to find parts of its inner coat destroyed by ulcerative inflammation; but that sort of decomposition of the stomach, mentioned by Mr. Hunter, as occurring in cases of sudden death, is extremely rare. Mr. Hunter's opinion, that this morbid appearance is caused by the gastric juice, cannot be accurate; for, except in a temperature approximating to that of the blood, it possesses scarcely any solvent power; and, even according to Mr. Hunter's own authority, the gastric juice cannot act on a substance endowed with vitality, of which the stomach continues to be possessed for hours after death.

